# Supplementary material for: Comparative multiomics analysis of cell physiological state after culture in a basket bioreactor
Source: Sci Rep. 2022 Nov 23;12:20161. doi: 10.1038/s41598-022-24687-4 (PMC9686226; doi:10.1038/s41598-022-24687-4)
Supplement: Supplementary file 1 — Supplementary Information 1. [file 41598_2022_24687_MOESM1_ESM.zip › raw data/Transcriptomic raw data/6.Enrichment/2.KEGG/TE1vsTC1/ALL/src/csab00970.html]

csab00970


- csab:103219751

- Up regulated genes

103219751(1.58279754803628)

- csab:103226274

- Up regulated genes

103226274(1.54777686795756)

- csab:103238568

- Up regulated genes

103238568(1.12646224416777)

- csab:103229694

- Up regulated genes

103229694(1.26611264939845)

Close
